# Pilot development with preliminary validation of a skills suturing score: essential suturing skills

**DOI:** 10.1007/s00464-026-13086-9

**Published:** 2026-07-13

**Authors:** Renato V. Soares, Marina S. Guérios, Fernanda Tsumanuma, Fabiana A. Andrade

**Affiliations:** 1 Postgraduate Program in Industrial Biotechnology, Positivo University, Curitiba, Brazil; 2 Positivo University, Curitiba, Brazil

**Keywords:** Surgical education, Suturing score, Surgical skills, Surgical competencies, Surgical training, Medical education

## Abstract

**Introduction:**

Suturing skills are paramount for all surgeons. To date, there is no unified validated score that correlates with basic suturing abilities. We sought to develop and validate a score (Essential Suturing Skills—ESS) to evaluate basic suturing performance.

**Methods:**

The ESS testing comprises two exercises. The applicant performs seven simple interrupted stitches in a dedicated foam base and follows with a running suture in a carpet-like material (seven throws). The time to perform the sutures and the surgical skills were compiled and combined through a novel mathematical equation, thus generating the ESS score. Fourth-year medical students (before and after 30 h of training), general surgery residents, and surgeons (control group) performed the ESS tasks. The evaluations were blinded. In the ESS score, a lower number indicates a better performance.

**Results:**

Sixty-two medical students (43 females), six general surgery residents, and eight surgeons have performed the ESS. The mean ESS score of the surgeons was 4.14 (± 0.45). The students had a 9.15 (± 2.10) ESS score before training and a 6.29 (± 0.98) after 30 h of training. The general surgery residents ESS score was (6.04 ± 0.91). The differences between the surgeons and students both before (*p* < 0.001) and after training were statistically significant (*p* < 0.0001). The improvement of the ESS score of the students after training was also significant (*p* < 0.0001). The residents had a similar performance compared with the students after training (*p* = 0.536) and significantly worse compared with the professors (*p* = 0.0002). Each evaluation took about 20 min to be completed.

**Conclusion:**

In this pilot validation study, the ESS score correlated well with suturing performance, with clear discrimination between medical students, residents, and surgeons. It is low cost and simple to perform and evaluate. Future developments include a pass/fail score, cognitive testing, and automated evaluation.

**Graphical abstract:**

**Figure:**
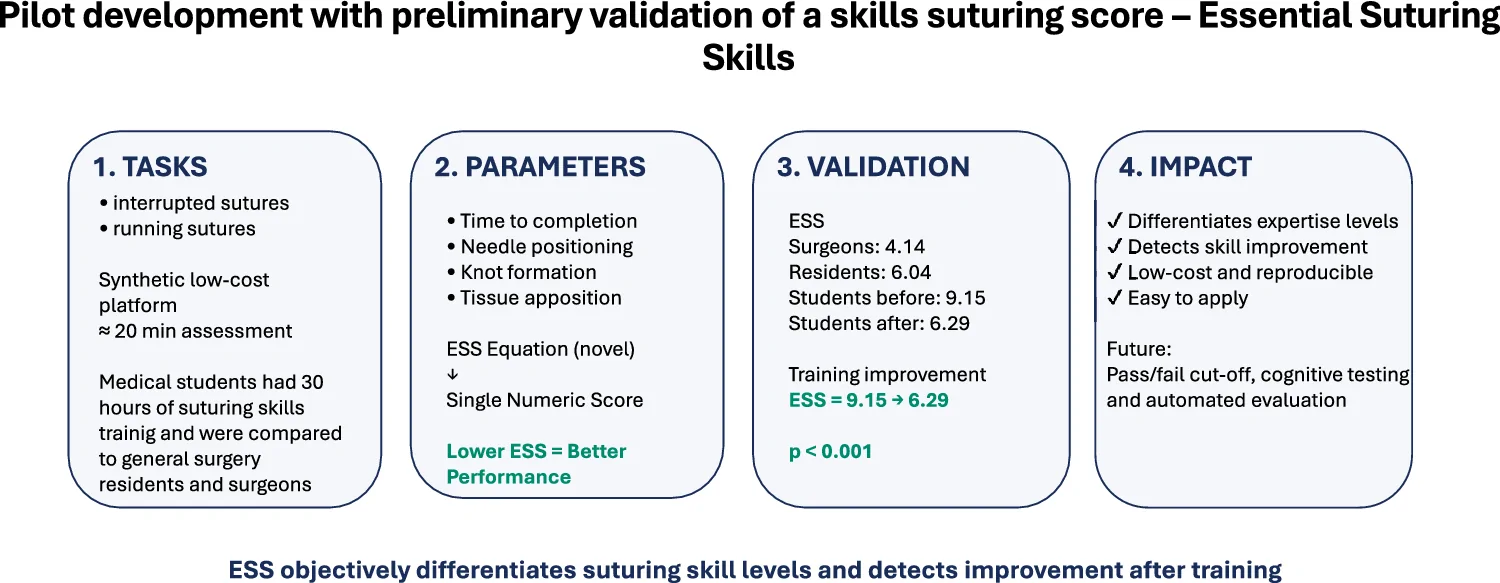

Suturing for repairing damaged tissues is an ancient method. Eyed needles, invented between 50,000 and 30,000 BC (Neolithic period), were likely used for sewing wounds. The famous Edwin Smith papyrus, the first known surgical text, was likely codified in 1600 BC in Egypt [1]. The first detailed description of surgical techniques and suturing was written by an Indian, Susruta in 500 BC. At first, the needles were handheld, but great refinement in surgical technique came as innovators such as Galen and Pare began to use instruments to hold and pivot the needles, along with the use of curved needles. The modern design of a needle holder came in the early twentieth century, along with the pronation and supination movements for throwing a stitch.

Properly performed surgical sutures are essential for successful surgery. For many years, surgical skills had been taught in the hospital environment, with patients being the learning models, using the see-do-teach model of Halsted [2]

Training in surgery has undergone intense transformation in recent decades. New technologies, such as laparoscopic and robotic surgery, as well as a myriad of new equipment, staplers and energy forms have been incorporated in surgical practice, with more content for those initiating in the surgical world to learn. Nonetheless, proper suturing skills remain paramount for all surgeons [2]. From an ethical and legal point of view, it is no longer acceptable that the entire learning curve for suturing to be performed on patients, as this leads to a significant increase in costs and worsening of the results of surgical interventions [3].

Competencies targeted certifications supported by SAGES (Society of American Gastrointestinal and Endoscopic Surgeons), such as the FLS (Fundamentals of Laparoscopic Surgery), FES (Fundamentals of Endoscopic Surgery), and FUSE (Fundamental Use of Surgical Energy), have been improving surgical education throughout the world. They serve well residents and surgical fellows [4–6]. Moving back toward initial surgical education, it is possible that a competence-based curriculum for suturing could serve junior residents and medical students as they first enter the operating theater and debut their surgical career.

A unified validated score that correlates with basic suturing abilities is still lacking, highlighting the need for a tool that can support this early stage of surgical training. In response to this gap, we sought to develop and validate a score (Essential Suturing Skills—ESS), based on suturing tasks performed in synthetic materials, to evaluate fundamental suturing performance.

## Methods and procedures

We conducted a prospective cohort study with forth-year medical students at Universidade Positivo, a private medical school in southern Brazil. In the first data collection, the students had already some very basic training in suturing. The ESS testing was performed by the students in the first and last lab sections of a Surgical Discipline in which they went about 30 h of training in basic surgical maneuvers, with suturing exercises being a large part of this practice. Surgical models included animal ex vivo parts, such as porcine foot, heart, tongue among others. A group of General Surgery residents (PGY1-3) and surgeons (which served as control group) performed the EES as well.

Before engaging in the EES tasks, the participants signed written informed consent, and the data were blinded and anonymized for analysis. EES score was analyzed for validation.

### ESS platform

A dedicated, low-cost, non-biological platform was developed for this study, using widely available and easy-to-build materials. The model is unique.

**Fig. 1:**
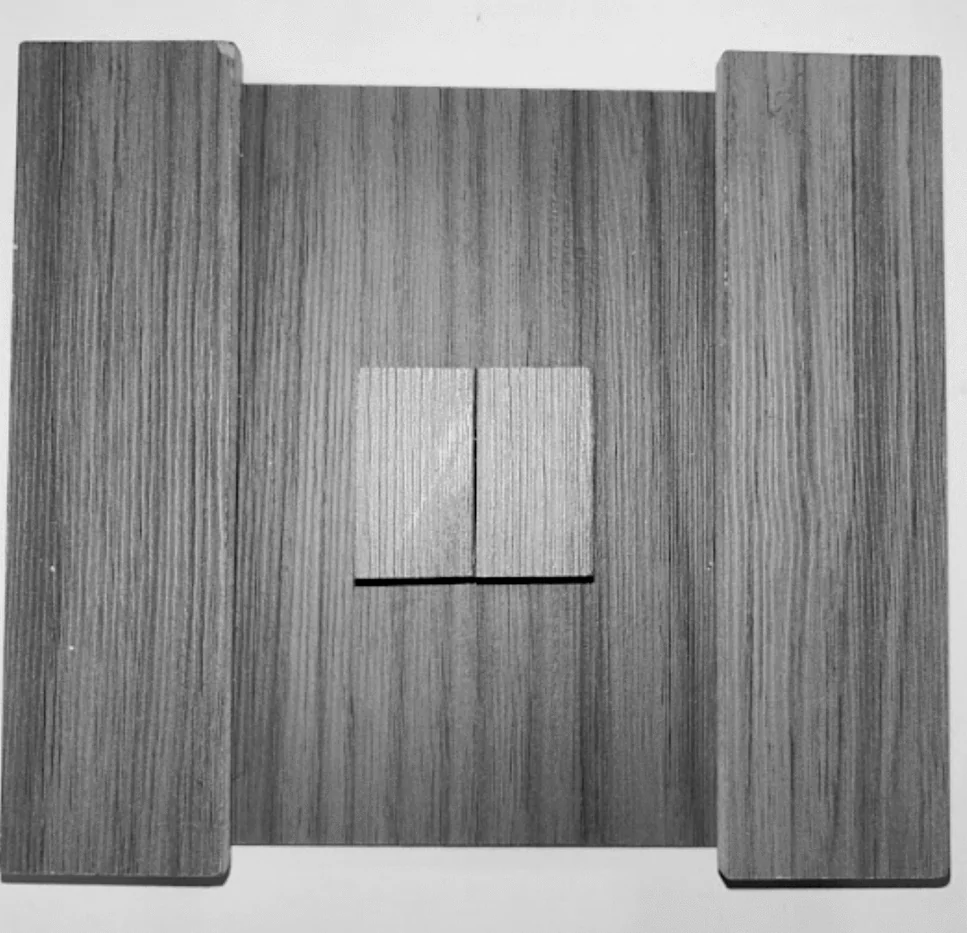
The handcrafted wooden base. It has an elevation in its middle part, simulating an open wound upon the accommodation of the foam or vinyl parts

It consists of a simple, handcrafted wooden base measuring 12 × 16 cm. On this base, we attached the two materials to be used, a simple foam with a density of 28 g/cm^3^ or a vinyl mat, depending on the activity to be performed (Fig. 1).

A regular basic suturing kit is used to complete the tasks. It comprised a 6 in needle holder, a 7.5 in straight Mayo scissors, a 6.25 in rat-tooth, and a 6.25 in grasping forceps (EDLO, Brazil).

### Description of ESS tasks

Applicants perform 2 basic tasks and are assessed according to scores freely adapted from the OSATS (objective structured assessment of technical skill) [7] combined with a novel mathematical equation, thus generating a numeric score ESS score (explained later in this section).

**Fig. 2:**
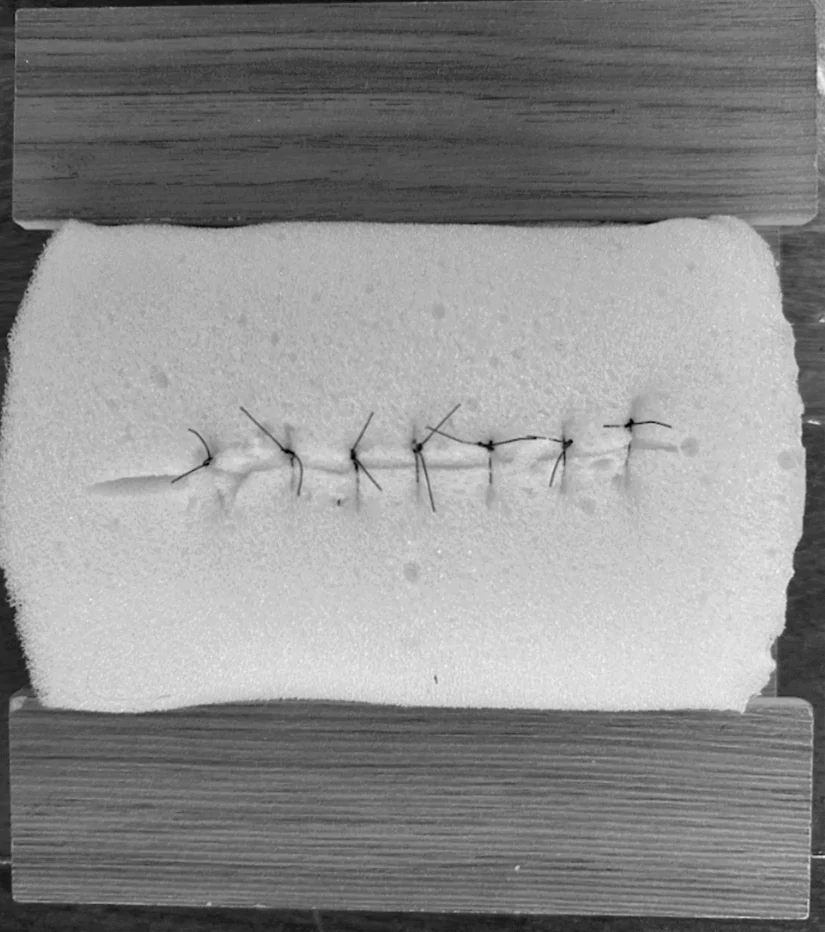
Sponge with horizontal simple interrupted sutures

First task -Simple interrupted suture—Seven simple interrupted horizontal stitches with 3–0 mononylon (3/8 20 mm reverse cutting needle, 45 cm-Medico (Huaian) Co., Ltd.—China, People’s Republic) (Fig. 2).

**Fig. 3:**
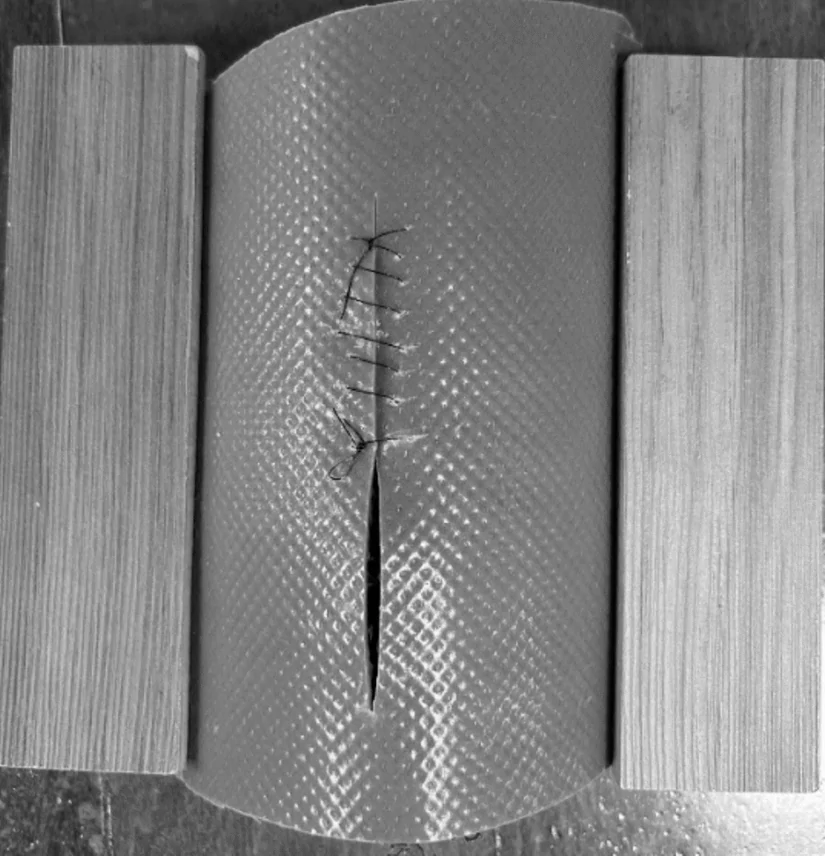
Vinyl mat with vertically placed running suture

Second task -Simple running suture—Seven throws simple running suture vertically with 3–0 mononylon (3/8 20 mm reverse cutting needle, 45 cm-Medico (Huaian) Co., Ltd.—China, People’s Republic) (Fig. 3).

### ESS score

1. The time to perform each task.
2. Assessment of suturing skill and technique, according to a 5-point Likert scale in the parameters below:
  2.1 Needle positioning in the needle holder (needle positioned correctly and at 90 degrees to the needle holder).

**Table:** 

1	2	3	4	5
Repeated attempts to position the needle without success		Often positions the needle correctly, with occasional errors		Correct needle placement on the first attempt
  2.2 Formation of surgical knots

**Table:** 

1	2	3	4	5
Surgical knots that are excessively loose or tight, do not perform in squares. Frequent breakage of the threads		Performs surgical knots with correct technique and tension in 50% of stitches, rare thread breaks		Performs surgical knots correctly without failures
3. Tissue apposition

**Table:** 

1	2	3	4	5
Asymmetrical grip of suture edges with inversion of the edge in most cases		Symmetrical and everted sutures in 50% of sutures		Symmetrical and everted sutures in 100% of cases

### Mathematical equation generating the ESS Score

To assess students’ performance in suturing skills, a composite score was developed integrating both objective measures (execution time) and subjective measures (skill evaluation) in two types of sutures: simple interrupted sutures (SS) and running sutures (RS). These components were combined through a mathematical equation, generating the ESS score, as follows: ESS = ((TSS/TSSS) + 15/PSS) + ((TRS/TRSS) + 15/PRS).

Here, TSS is the individual time obtained by the student in the simple interrupted suture activity; TSSS is the mean execution time of simple interrupted sutures obtained from surgeons (reference standard); PSS is the score obtained by the student in the practical assessment of simple interrupted sutures (0–15 points); TRS is the individual time obtained in the running suture activity; TRSS is the mean execution time of running sutures obtained from surgeons; and PRS is the score obtained by the student in the practical assessment of running sutures (0–15 points).

The equation was designed to integrate two essential aspects of performance: efficiency (time component), represented by the ratio between the student’s execution time and the surgeons’ mean time, where ratios close to 1 indicate performance similar to the reference standard; and technical quality (score component), which reflects the relationship between the maximum possible score (15 points) and the score obtained by the student.

The components of both suture modalities (SS and RS) are then summed, generating a global score in which lower values indicate better overall performance.

### Statistical analysis

Statistical analyses were performed to compare ESS scores between groups and across training periods. Continuous variables were expressed as mean ± standard deviation (SD). Comparisons between independent groups (surgeons and residents) were performed using Student’s *t* test. Paired comparisons between pre-training and post-training were analyzed using the paired Student’s *t* test. A significance level of 5% (*p* < 0.05) was adopted for all analyses. Validity evidence for the ESS score was interpreted according to the Messick framework for assessment validation [8, 9]. The internal consistency of the instrument was assessed using Cronbach’s alpha coefficient for each suturing technique (simple interrupted and simple running sutures) and for each assessment moment (pre- and post-training). Cronbach’s alpha values were interpreted according to commonly accepted thresholds, with values ≥ 0.70 considered acceptable, ≥ 0.80 good, and ≥ 0.90 indicative of excellent internal consistency [8, 9]. Given the small number of items in each scale, the average inter-item correlation was also calculated as a complementary measure of internal consistency. Inter-item correlations were obtained using Pearson’s correlation coefficient, and the mean correlation was computed across all item pairs within each scale. Values between 0.20 and 0.40 were considered indicative of adequate internal consistency for short scales [10].

## Results

**Fig. 4:**
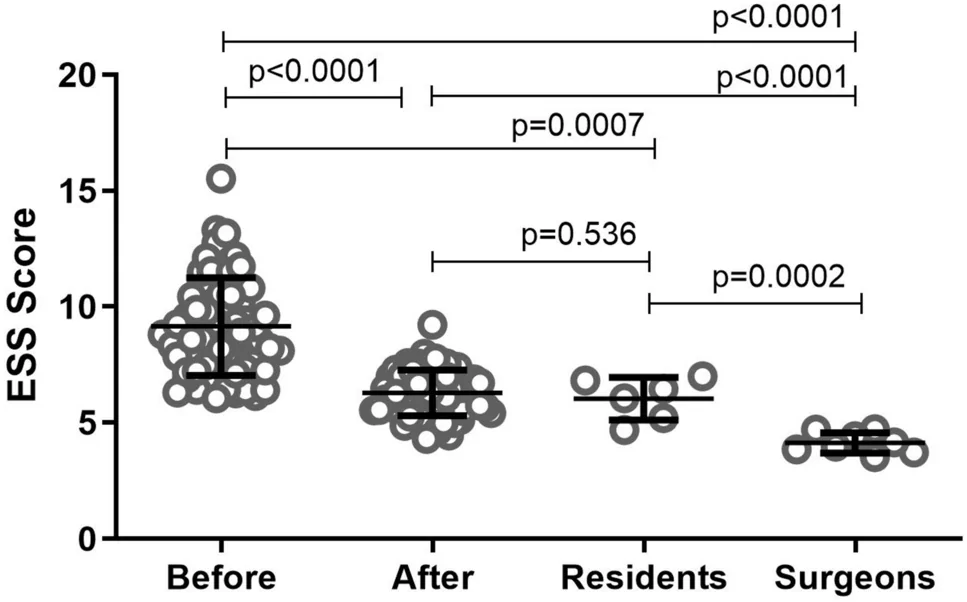
Comparison between ESS scores of students before and after training, residents and surgeons

Sixty-two medical students (43 females), six general surgery residents (PGY1-3), and eight surgeons have performed the ESS. The time to perform the tasks and the quality of the suturing is outlined in Table 1. The mean ESS score of the surgeons was 4.14 (± 0.45). The students had a 9.15 (± 2.10) ESS score before training and a 6.29 (± 0.98) after 30 h of training. The residents had a ESS score of 6.04 (± 0.91). The differences between the surgeons and students both before training (*p* < 0.0001) and after training were statistically significant (*p* < 0.0001). The improvement of the ESS score of the students after training was also significant (*p* < 0.0001—Fig. 4). The residents had a trend toward better performance compared to the students after training (*p* = 0.536), but significantly worse compared to the surgeons (*p* = 0.0002). Each evaluation took about 20 min to be completed.

**Table 1:** Skills and performance of students, residents, and surgeons and the unified ESS score

	Interrupted sutures		Running sutures		EES score
	Time (min:s)	Skills score	Time (min:s)	Skills score	
Surgeons (*n* = 8)*	03:46	15.0	02:20	13.67	4.14
Residents (*n* = 6)*	04:59	11.7	04:46	11.2	6.04
Students before (*n* = 62)*	08:25	9.16	06:40	8.60	9.15
Students after (*n* = 62)*	05:41	13.35	05:51	13.21	6.29

* represents Mean

### Preliminary validity of the ESS analysis

Objective evaluation of very basic surgical skills is a difficult task. The authors of this study have been confronted with this assignment for years, as professors of surgery teaching unskilled medical students.

In a well populated and developing country such as Brazil, many third- and fourth-year’s medical students are taking internships in hospitals outside the University and performing basic suturing under supervision, especially in emergency rooms.

The basic question to be answered is if these students have actually acquired the competence of suturing, before they attempt to do so in a patient. Over the years, we have been refining evaluation methods in order to be realistic and fair as we test student’s abilities. However, a standardized way of measuring one’s suturing skills is lacking. Thorough discussions among experts along with a literature review had led us to this first version of the ESS. In the following session, we went over the Messick validation framework to discuss the different aspects of validity of this innovative suturing score system [11, 12].

### Content validity

The tasks of the ESS aim to replicate basic suturing in a real scenario. The most commonly performed types of suturing in clinical practice are simple interrupted and simple running [13, 14]. Tissue stiffness may be an issue for the beginner. We simulated this by taking the two tasks of the test (simple interrupted and simple running) in materials of different consistencies. While the foam simulated a more pliable tissue (such as fat or soft skin), the vinyl mat simulated harder tissue (such as fascia). While these materials can be considered low fidelity (not very similar to human tissues), it was demonstrated that model fidelity does not interfere with suture acquisition skills [15]. We chose monofilament 3–0 nylon threads due to its low cost and wide availability and because it is the most used to skin closure [13, 16]. Besides, we tested vertical and horizontal suturing skills, since the first task simulated a horizontal wound and the second a vertical one. Finally, we adapted published assessment studies [7, 17–20] that used domains such as tissue apposition, knot formation, and needle positioning as correlates of adequate suturing technique, along with the time to perform the tasks [4, 18].

### Response process

In the ESS, we need an individual evaluator for each applicant. In our case, professors of surgery were the evaluators. The senior author designed a series of short videos (1–5 min duration) explaining how to evaluate and score the applicants. All the evaluators have large experience in grading students and saw the explanatory videos. The ESS was pilot tested in two different occasions, first with 06 applicants and the second time with 30 applicants (unpublished data). Errors were corrected and the definitive format was assembled.

The applicants were told to use one suture tread of 3–0 nylon for each task. In case they needed a second tread, it was provided without change of the score.

An electronic form was filled by the evaluator as the applicants performed the ESS. We were not able to test inter-evaluator variation, which is a future step in the project. The evaluators did not know the final ESS score. All the applicants had different evaluators the first and second time they performed the ESS. The time required to accomplish the ESS was about 20 min per applicant.

### Internal structure

The internal consistency of the instrument differed substantially between the pre- and post-training assessments. Before training, Cronbach’s alpha was high across items in both the simple interrupted suture and simple running suture evaluations (*α* = 0.955 and 0.944, respectively), indicating strong correlations among items. After training, Cronbach’s alpha decreased (*α* = 0.473 and 0.375, respectively). Despite this reduction, the average inter-item correlation remained within the acceptable range for short scales (*r* = 0.23 and 0.20, respectively), suggesting adequate coherence among the evaluated items. The high internal consistency observed before training likely reflects homogeneous performance across items and limited discrimination between specific components of surgical skill. In contrast, the reduction in Cronbach’s alpha after training may indicate increased differentiation among skill domains, as participants began to exhibit more heterogeneous performance profiles across needle positioning, knot tying, and tissue apposition, as well as between different suturing techniques. This finding is consistent with the multidimensional nature of surgical skill acquisition, in which distinct competencies may develop at different rates.

Importantly, Cronbach’s alpha is influenced not only by the degree of inter-item correlation but also by the number of items in the scale, tending to underestimate reliability in instruments with few items and assuming unidimensionality of the construct [8, 9]. Therefore, the lower post-training alpha does not necessarily represent a limitation of the instrument, but rather suggests improved sensitivity in capturing specific aspects of technical performance. The acceptable average inter-item correlation observed supports this interpretation [10], indicating that the items remain sufficiently related while measuring complementary dimensions of surgical skill.

### Relationship to other variables

In this pilot validation of the ESS, we tested four groups expected to be in different level of skills. The first group tested (students before training, *N* = 62) consisted of 4th year medical students who had already basic instructions in suturing and about 5 h of practical training. In this group, the ESS captured well the expected heterogenicity and the high ESS scores (9.15 ± 2.10), correlated with poor performance. This same group, tested blindly after 30 h of training, became more homogeneous and significantly improved the ESS scores (mean 6.29). We also tested six general surgery residents (PGY1-3), trending toward a better performance in comparison with the students after training, which also would be expected. The eight surgeons tested had a better performance compared with all other groups, including the residents, demonstrating the discriminating power of the ESS.

### Consequences of testing

The ESS has the aim of attesting the competence of suturing. As such, a high ESS score should guide the need for additional training before clinical exposure, for medical students and junior residents. Encouraged by the excellent initial psychometric performance of the ESS, the authors are gathering more data in order to establish a pass/fail score. The ESS requires minimal logistics, is low cost, and fast, thus facilitating widespread implementation.

## Discussion

Motor skills are acquired in three stages [21]. In the early (cognitive) phase, the beginner tries to understand the task. In this phase, instructions and cues are most valuable. The second (associative) phase is when the learner works to correct mistakes and perform the task more fluidly without interruptions. Deliberate practice and feedback are the cornerstone [22]. In the third (autonomous) phase, the speed and efficiency of performance continue to improve, although at a slower pace. Long hours of dedication and diligent training may lead a few professionals to an Expert performance level, as defined by Ericsson [23]. In our ESS testing study, we measured students in the early cognitive phase before training, and a wide variation in performance was captured. In practical terms, one could assume that ESS can help differentiate the students with more prominent motor skills from those who would demand more attention from the instructor, thus preventing further trouble in the learning process. With 30 h of guided practice over 20 weeks, the students presumably evolved to the associative or autonomous phase, and the improvement was well captured by the ESS score. Interestingly, even after training, the students have not reached the surgeons level, demonstrating again the discriminant power of the ESS. For a task as simple as stitching, we presumed that the ceiling of the score could be reached by the students after training, but the results demonstrated otherwise.

A number of methods for assessing surgical skills have been developed. The OSATS (Objective Structured Assessment of Technical Skill) [24], first published in 1997, describes a series of tasks performed by surgical trainees, simulating discrete steps in operative procedures in general surgery, such as bowel and vascular anastomosis. The trainees were evaluated by a qualified surgeon using a checklist and a score was generated. OSATS framework has been adapted to other surgical specialties such as Gynecology [25] and Dermatology [26], and vast literature demonstrates its validity [17, 26, 27]. In the original description [24], the test had eight tasks and took two hours to complete. The Multiple Objective Measures of Skill (MOMS) aimed to assess the skills of basic surgery trainees, with 6 (one cognitive and 5 motor) tasks taking about 90 min to be completed, with the evaluation using an OSATS scale [28]. In comparison, the ESS testing takes only 20 min to complete and requires simple materials, with minimal logistics and costs. For laparoscopic surgery, the Society of American Gastrointestinal and Endoscopic Surgeons (SAGES) created the FLS program, aiming to improve surgical education through a structured curriculum, with cognitive and psychomotor parts. Since 2009 the FLS is required for General Surgery graduating residents [4].

In the ESS, there is no cognitive testing at all, which is under development. Also, we are gathering more data so we can have a pass/fail score in the ESS. That could work as an indicator for the competency of suturing. As many preceptors see the FLS testing as a basic requirement to allow a resident to perform laparoscopy, we can imagine a minimal ESS scoring as a requirement to let a medical student or a resident stitch a patient or scrub for a case.

To date, the ESS testing requires an evaluator. As a pilot validation project, we had one researcher measuring the performance of each student. The first and second evaluations of the study group were performed by two different evaluators, blinded to the results of the first evaluation, and we were not able to measure the inter-observer consistency in this project. Along with this next step, we are also envisioning an automated system based on artificial intelligence (AI) and deep learning, so the practitioner can do the test multiple times and have validated feedback. For complex surgeries such as colectomies and cholecystectomies, AI-based skill assessments are under evaluation [29].

## Conclusion

In this pilot validation study, the ESS score correlated well with suturing performance, with clear discrimination between medical students, residents, and surgeons. It is low cost and simple to perform and evaluate. Future developments include a pass/fail score, cognitive testing and automated evaluation.
